# Mutational Analysis of Mitochondrial tRNA Genes in Patients with Asthma

**Published:** 2017-05

**Authors:** Chun Mei WANG, Xiao Jing ZHANG, Ying Jun MA, Xia LI

**Affiliations:** 1. Bright New District Shenzhen City, Guangdong Province People’s Hospital, Shenzhen, Guangdong, China; 2. Dept. of Pathology, Medical College, Shenzhen University, Shenzhen, Guangdong, China

**Keywords:** Asthma, Children, Mitochondrial tRNA, Mutation, Pathogenicity, China

## Abstract

**Background:**

Mitochondria are autonomous cellular organelles that oversee a variety of functions such as metabolism, energy production, calcium buffering, and cell fate determination. Most recently, mitochondrial dysfunction caused by mitochondrial mutations played important roles in the pathogenesis of asthma. However, the frequency of mitochondrial tRNA mutations in asthma is largely unknown.

**Methods:**

Overall, 200 patients with asthma and 100 healthy control subjects were recruited between Jan 2015 and Dec 2015 at the Guangming New District People’s Hospital, Shenzhen, Guangdong Province, China. We first performed PCR amplification of the mitochondrial tRNA genes and subsequently sequenced the PCR products, and we used the pathogenicity scoring system to evaluate the potential role of these mutations.

**Results:**

Two patients carrying the tRNA^Thr^ G15927A mutation, three patients carrying the tRNA^Ala^ T5655C mutation and one patient carrying the tRNA^Glu^ A14693G mutation, these mutations were absent in healthy controls. Moreover, these mutations located at positions highly conserved between different species, and may cause a failure in mitochondrial tRNA metabolism, consequently result in mitochondrial dysfunction that responsible for asthma. In addition, the pathogenicity scoring system showed that these mutations should be regarded as “pathogenic”.

**Conclusion:**

Mitochondrial tRNA mutations caused the mitochondrial dysfunction may be involved in the pathogenesis of asthma. Thus, this study provided novel insight into the molecular mechanism underlying mitochondrial tRNA mutations in asthma. Moreover, screening for the mitochondrial tRNA mutations was advised for the diagnosis of patients with asthma.

## Introduction

Asthma is a chronic inflammatory disorder of the lungs that causes intermittent airway obstruction, increased airway hyper-responsiveness and recurrent respiratory symptoms such as wheezing, breathlessness, chest tightness and coughing (1). To date, the molecular mechanism underlying this disease remains poorly understood. Many environmental stimuli are known to further its development like, for example, exposition to cigarette smoke or certain allergens. Although genetic contributions from the father are important, particularly regarding airway hyper-responsiveness, maternal history of asthma more strongly influences development of asthma (2). Data from several prospective birth cohorts suggest that a maternal (but not paternal) history of asthma confers substantial risk for development of persistent wheeze or asthma (3). Since mitochondria are inherited through the maternal line, it raises the possibility that the mitochondrial dysfunction may contribute to the pathogenesis of asthma.

Mitochondria are the “cellular powerhouses,” which generate most of a cell’s ATP through oxidative phosphorylation (OXPHOS). Human mitochondrial DNA (mtDNA) encodes 13 essential polypeptides of the OXPHOS system, as well as 2 rRNAs and 22 tRNAs for mitochondrial translation. Mutations in mtDNA have been implicated to be associated with a wide range of clinical disorders such as deafness (4); Leber’s Hereditary Optic Neuropathy (5) and hypertension (6). Most recently, the haplogroup (common mitochondrial polymorphisms) U has been shown to be associated with total serum IgE levels in asthmatics (7), highlights the importance of mitochondrial genome mutations in asthma. With the purpose of understanding the role of mitochondrial mutations in asthma, we carried out a systematic mutational analysis of mitochondrial tRNA (mt-tRNA) genes in 200 asthma infants and 100 controls, in this study, PCR-Sanger sequencing showed the presence of three known mt-tRNA mutations.

## Materials and Methods

### Subjects

Overall, 200 infant patients with bronchial asthma (45% males and 55% females, aged 3–5 yr) were recruited between Jan 2015 and Dec 2015 at the Guangming New District People’s Hospital, Shenzhen, Guangdong Province, China. Moreover, 100 unrelated healthy controls with the age and gender matched were collected in the same area. An extensive medical history was recorded in all patients including previous occurrence and duration of wheezing symptoms, acute medications, severity of asthma attacks, symptoms of allergic rhinitis or conjunctivitis, atopic dermatitis, and any family history of allergic diseases. The Ethical Committee of the Guangming New District People’s Hospital approved blood and experimental procedures. A statement of informed consent was signed by the parents of all participating individuals.

### Screening for the mt-tRNA mutations

We used the primers for genetic amplification of the 22 mt-tRNA genes, the information of the primers were listed in Table 1.

**Table 1:** Primers for PCR amplification of the mt-tRNA genes

**Target gene**	**Primer name**	**Primer Sequence (5’–3’)**	**Product size**
tRNA^Phe^	MT-1F	CTCCTCAAAGCAATACACTG	802 bp
	MT-1R	TGCTAAATCCACCTTCGACC	
tRNA^Val^	MT-2F	CGATCAACCTCACCACCTCT	802 bp
	MT-2R	TGGACAACCAGCTATCACCA	
tRNA^Leu(UUR)^	MT-4F	AAATCTTACCCCGCCTGTTT	887 bp
	MT-4R	AGGAATGCCATTGCGATTAG	
tRNA^Ile^	MT-6F	TGG CTC CTT TAA CCT CTC CA	898 bp
tRNA^Gln^			
tRNA^Met^	MT-6R	AAG GAT TAT GGA TGC GGT TG	
tRNA^Ala^	MT-8F	CTAACCGGCTTTTTGCCC	814 bp
tRNA^Asn^			
tRNA^Cys^	MT-8R	ACCTAGAAGGTTGCCTGGCT	
tRNA^Ser(UCN)^	MT-11F	ACGCCAAAATCCATTTCACT	987 bp
tRNA^Asp^	MT-11R	CGGGAATTGCATCTGTTTTT	
tRNA^Lys^	MT-12F	ACG AGT ACA CCG ACT ACG GC	900 bp
	MT-12R	TGG GTG GTT GGT GTA AAT GA	
tRNA^Gly^	MT-15F	TCTCCATCTATTGATGAGGGTCT	891 bp
tRNA^Ar**g**^	MT-15R	AATTAGGCTGTGGGTGGTTG	
tRNA^His^	MT-18F	TATCACTCTCCTACTTACAG	866 bp
tRNA^Ser(AGY)^			
tRNA^Leu(CUN)^	MT-18R	AGAAGGTTATAATTCCTACG	
tRNA^Glu^	MT-21F	GCATAATTAAACTTTACTTC	938 bp
	MT-21R	AGAATATTGAGGCGCCATTG	
tRNA^Thr^	MT-22F	TGAAACTTCGGCTCACTCCT	1162 bp
tRNA^Pro^	MT-22R	GAGTGGTTAATAGGGTGATAG	

We first extracted the genomic DNA from each sample, using the Puregene DNA Isolation kit (Gentra Systems, Minneapolis, MN, USA). The PCR primers were supplied by BGI (Shenzhen, China) and the PCR mixture included 200 μm dNTP, 10X buffer, Taq DNA polymerase and 15 mmol/L Mg^2+^ (Takara Biotechnology Co., Ltd., Dalian, China). Each amplified DNA sample was purified and analyzed using the ABI 3700 automated DNA sequencer and the Big Dye Terminator Cycle sequencing reaction kit (Applied Bio-systems; Thermo Fisher Scientific, Waltham, MA, USA). The sequence data were compared with the reversed consensus Cambridge sequence to screen the mutations (GenBank Accession No. NC_012920) (8).

### Pathogenicity scoring system for these mt-tRNA mutations

A program was provided for assigning a pathogenicity score to mt-tRNA mutations (9). Their weighting scoring system was revised in 2011 (10). According to that standard, we classified a mutation as “neutral polymorphism” with a score ≤6, whereas the score was ranking from 7–10, it belonged to “possible pathogenic”, if the score μ11, it belonged to “definitely pathogenic”.

## Results

### Mutational analysis of mt-tRNA genes

**Fig. 1:**
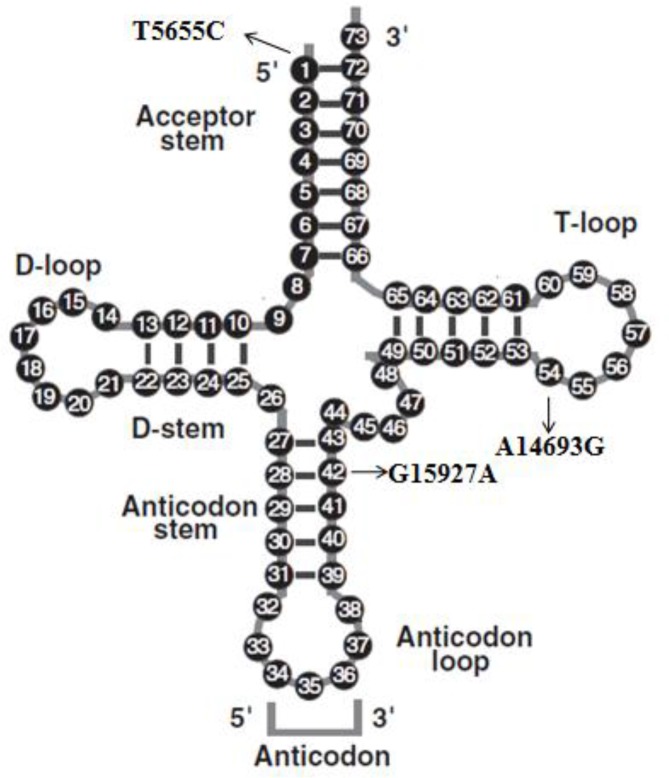
Cloverleaf structure of mt-tRNA with standard nucleotide numbering, arrows indicate the positions of 1, 42 and 54, corresponding to the T5655C, G15927A, and A14693G mutations

Mutational analysis of the 22 mt-tRNA genes led us to identify 3 mutations: tRNA^Thr^ G15927A; tRNA^Ala^ T5655C and tRNA^Glu^ A14693G. Of these, the G15927A mutation was detected in 2 out of 200 asthmatic pediatric patients (1%), the T5655C mutation was detected in 3 patients (1.5%) and the A14693G mutation was detected in 1 patient (0.05%). We failed to detect any mt-tRNA mutations in control subjects, moreover, all these mutations were not identified in healthy controls; the location of each mt-tRNA mutation was displayed in Fig. 1.

### Determining the pathogenicity

According to the pathogenicity scoring system (9,10), the total scores of the G15927A, T5655C and A14693G mutations were 11, 11 and 9 points, respectively (Table 2). Thus, the G15927A and T5655C mutations should be regarded as “definitely pathogenic”, while the A14693G mutation should be classified as “possibly pathogenic”.

**Table 2:** The pathogenicity scoring system for the mt-tRNA mutations

**Scoring criteria**	**G15927A mutation**	**Score/20**	**T5655C mutation**	**Score/20**	**A14693G mutation**	**Score/20**	**Classification**
More than one independent report	Yes	2	Yes	2	Yes	2	
Evolutionary conservation of the base pair	No changes	2	No changes	2	No changes	2	≤6 points: neutral polymorphisms; 7∼10 points: possibly pathogenic; ≥11 points: definitely pathogenic
Variant heteroplasmy	No	0	No	0	No	0	
Segregation of the mutation with disease	Yes	2	Yes	2	Yes	2	
Histochemical evidence of mitochondrial disease	No evidence	0	No evidence	0	No evidence	0	
Biochemical defect in complex I, III or IV	No	0	No	0	No	0	
Evidence of mutation segregation with biochemical defect from single-fiber studies	No	0	No	0	No	0	
Mutant mt-tRNA steady-state level or evidence of pathogenicity in transmitochondrial cybrid studies	Yes	5	Yes	5	Weak evidence	3	
Maximum score	definitely pathogenic	11	definitely pathogenic	11	possibly pathogenic	9	

## Discussion

In this study, we screened the potential pathogenic mt-tRNA mutations with asthma. Asthma is the result of the interaction of multiple genetic and environmental factors (1). Multiple genes may be involved in the pathogenesis of asthma (11). However, these genes are mainly nuclear genes, recently; the role of mitochondria in asthma pathogenesis has received considerable attention.

“Since mitochondria use OXPHOS to convert dietary calories into usable energy, releasing reactive oxygen species (ROS) as a toxic by-product. A significant number of epidemiological and clinical studies support the relationship between increased ROS and the pathogenesis of bronchial asthma (12). Moreover, gene encoding tRNA is the hotspot for pathogenic mutations associated with human mitochondrial diseases. Up to date, over 150 different pathogenic mutations have been reported located within mt-tRNA genes (13). As most of the mitochondrial proteins, are nuclear, encoded and mt-tRNAs act as key effectors in translation and linked to metabolic activity. Thus, drove us to analysis the mt-tRNA mutations in children with asthma. Moreover, five mitochondrial tRNA variants were identified in 76 asthmatic patients, tRNA^Leu(CUN)^ A12308G, tRNA^Phe^ 595insC, tRNA^Thr^ G15928A, tRNA^Lys^ A8343G, tRNA^Arg^ T10448C variants are implicated to be associated with asthma (14). Sequence analysis of the mt-tRNA genes revealed the presence of three mutations: G15927A, T5655C, and A14693G. The homoplasmic G15927A mutation was localized at the fourth base in the anticodon stem of tRNA^Thr^ (15) (Fig. 1). A guanine at this position was conserved highly from bacteria to human mitochondria (16) and disrupted the 28C-42G base pairing. Functional characterization of cell line carrying the G15927A mutation showed a marked decreasing in the level of tRNA^Thr^ (17). While the homoplasmic T5655C mutation occurs at the 3’ end (at position 1) of the tRNA^Ala^. This nucleotide may act as a discriminator responsible for the identity of most tRNAs and plays an important role in the recognition by their cognate aminoacyl-tRNA synthetase (18). Thus, this mutation may cause a defect in the pre-tRNA processing, reduced the steady-state level of tRNA^Ala^ (19). In addition, the homoplasmic A14693G mutation occurs at the extremely conserved nucleotide of tRNA^Glu^ (position 54) (16) and is implicated to be associated with MELAS (mitochondrial encephalomyopathy, lactic acidosis, and stroke-like episodes) and to influence the phenotypic expression of deafness-associated 12S rRNA A1555G mutation (20, 21). In fact, nucleotide at position 54 is often modified; thereby contributing to the structural formation and stabilization of functional tRNAs. Mutations in mt-tRNAs can be either pathogenic or neutral polymorphism. Several attempts have been made to identify the criteria for pathogenic mutations, summarized as location in evolutionarily conserved sites, primarily in the stem structures with the disruption of Watson-Crick base pairing (9). Using the pathogenicity scoring system, all these mt-tRNA mutations should be classified as “pathogenic”, as they reached a high score ≥ 6 points.

We proposed the molecular mechanism underlying mt-tRNA mutations in asthma might be as follows: first, the mutation itself disrupts the secondary structure of mt-tRNA and subsequently results in a failure in mt-tRNA metabolisms such as the CCA addition, post-transcriptional modification and aminoacylation (22). Excessive ROS will subsequently damage the mitochondria until apoptosis of bronchial epithelial cells occurs. In this way, allergic inflammation of the bronchial epithelial cells can lead to dysfunction and remodeling of the airways during the course of asthma.

## Conclusion

Mt-tRNA mutations may play important roles in the pathogenesis of asthma; screening for the common mt-tRNA mutations is advised for the diagnosis of children with asthma.

## Ethical considerations

Ethical issues (Including plagiarism, informed consent, misconduct, data fabrication and/or falsification, double publication and/or submission, redundancy, etc.) have been completely observed by the authors.
